# Emergence and Reemergence of Porcine Reproductive and Respiratory Syndrome Virus (PRRSV)

**DOI:** 10.2174/1874357901711010048

**Published:** 2017-06-30

**Authors:** Kegong Tian, Xiangdong Li

**Affiliations:** 1 OIE PRRS Reference Laboratory, Beijing, China; 2 Henan Agricultural University, Zhengzhou, China; 3 National Research Center for Veterinary Medicine, Luoyang, China

PRRSV has obsessed world pig industry for decades though the commercial vaccines and stamping-out policy have been widely used in many countries. So far, PRRSV is still prevalent in North America and most Asian countries and leads to huge economic losses. PRRSV is notorious for its quick rate of mutation and recombination that may lead to the change of virulence. A good example is the emergence of highly pathogenic PRRSV (HP-PRRSV) in 2006 that led to huge amount of economic losses in Chinese pig industry.

 Recently, the emergence of NADC30-like PRRSVs that showed the highest similarity with NADC30, a North American type 2 PRRSV which has been isolated in United States of America in 2008, were draw much attentions from PRRSV researchers due to the fact of widespread of them in vaccinated pig herds. Therefore, the emergence and reemergence of PRRSV has huge impact on pig industry worldwide and reasonable control programs including vaccination will help to improve current situation. In this special issue of by *The Open Virology Journal*, the most recent epidemiology, serology, vaccinology and biosecurity measurements of PRRSV dedicated by the leading international research groups in PRRSV field will be included. 

